# Protection of navy-bean bioactive peptides within nanoliposomes: morphological, structural and biological changes

**DOI:** 10.1186/s40643-023-00709-5

**Published:** 2023-12-01

**Authors:** Nazila Zeynali Namdar, Leila Roufegarinejad, Ainaz Alizadeh, Narmela Asefi, Seid Mahdi Jafari, Khashayar Sarabandi

**Affiliations:** 1 Department of Food Science and Technology, Islamic Azad University, Tabriz Branch, Tabriz, Iran; 2 Department of Food Materials & Process Design Engineering, Gorgan University of Agricultural Sciences and Natural Resources, Gorgan, Iran; 3 Research Institute of Food Science and Technology (RIFST), Km 12 Mashhad-Quchan Highway, PO Box: 91895-157-356, Mashhad, Iran

**Keywords:** Navy-bean peptides, Nanoliposomes, Biological activity, Structural properties, Chemical stability

## Abstract

This study aimed to produce bioactive peptides from navy-bean protein with alcalase and pepsin enzymes (30–300 min) and to load them into a nanoliposome system to stabilize and improve their bioavailability. The degree of hydrolysis and biological activities (scavenging of DPPH, OH, and ABTS free radicals, reducing power, and chelating metal ions) of navy-bean protein were affected by the type of enzyme and hydrolysis time. The average particle size (83–116 nm), PDI (0.23–0.39), zeta potential (− 13 to − 20 mV), and encapsulation efficiency (80–91%) of nanoliposomes were influenced by the type and charge of peptides. The storage temperature and the type of loaded peptide greatly affected the physical stability of nanocarriers and maintaining EE during storage. The FTIR results suggested the effect of enzymatic hydrolysis on the secondary structures of protein and the effective placement of peptides inside polar-regions and the phospholipid monolayer membrane. SEM images showed relatively uniform-sized particles with irregular structures, which confirmed the results of DLS. The antioxidant activity of primary peptides affected the free radical scavenging of loaded nanoliposomes. Liposomes loaded with navy-bean peptides can be used as a health-giving formula in enriching all kinds of drinks, desserts, confectionery products, etc.

**Graphical Abstract:**

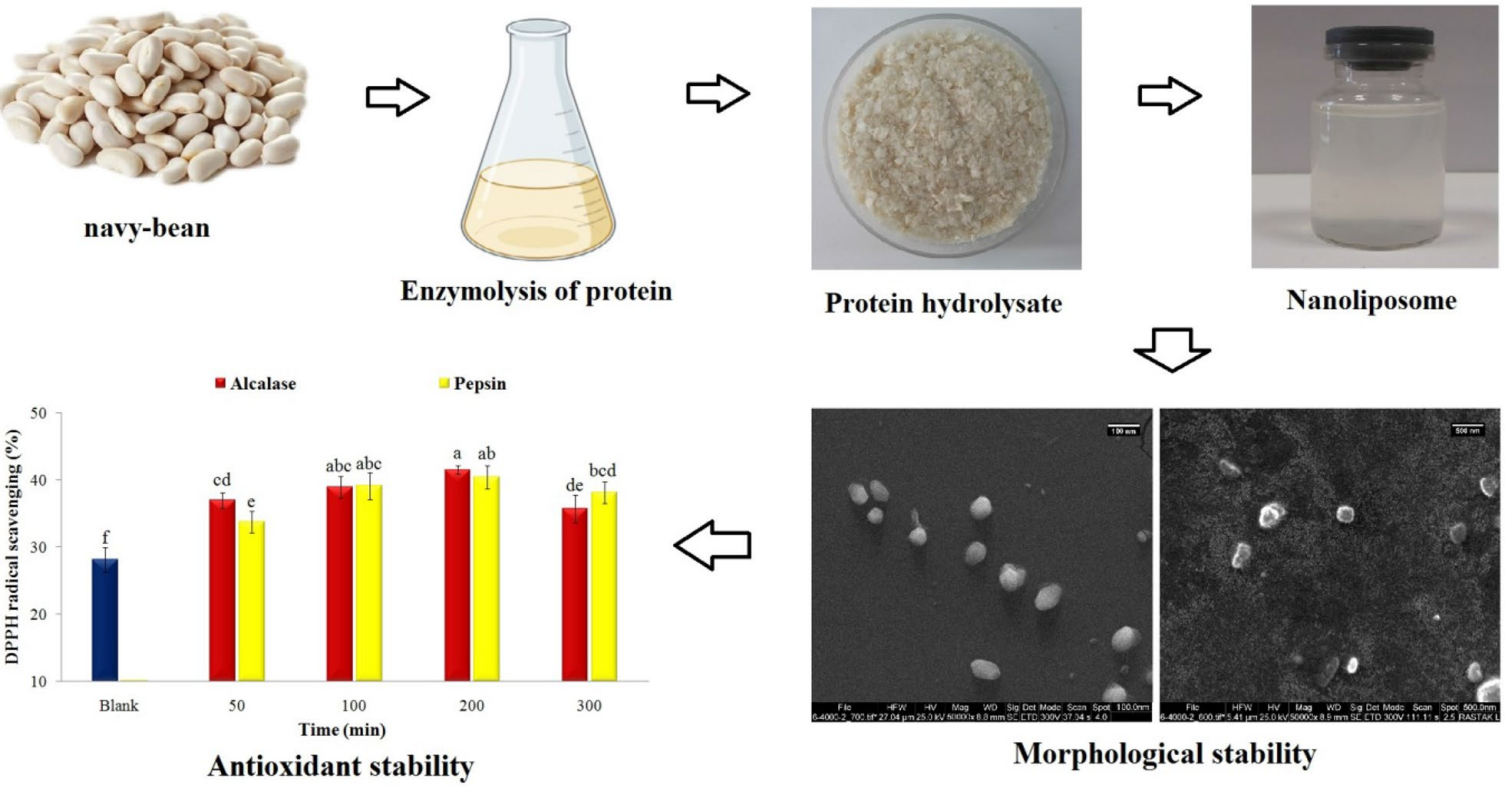

**Supplementary Information:**

The online version contains supplementary material available at 10.1186/s40643-023-00709-5.

## Introduction

In recent years, numerous biological activities of bioactive peptides have encouraged researchers in the food and pharmaceutical industries to pay special attention to these compounds (Akbarbaglu et al. 2022), which are sequences containing 2–20 amino acids (AAs) buried in the primary protein structure. Chemical methods, fermentation, and enzymatic hydrolysis (enzymolysis) are mainly used to release these parts (Islam et al. 2021). The most important health-promoting properties of bioactive peptides (from various plant, animal and marine sources) include their high digestibility, low allergenicity, antioxidant, anti-diabetic, antibacterial, anticancer, antithrombotic, opiate-like, immunomodulatory, hypertensive, and hypocholesterolemic activities (Bhandari et al. 2020). These advantages have resulted in evaluating the potential of various plants, animals, and marine sources for the extraction and production of bioactive peptides (Chakrabarti et al. 2018). These studies include enzymolysis and production of bioactive peptides from flaxseed (Sarabandi and Jafari 2020a), whey (Mohan et al. 2018), rainbow trout skin (Ramezanzade et al. 2017), salmon protein (Li et al. 2015), walnut (Moghadam et al. 2020), *Arthrospira platensis* (Akbarbaglu et al. 2023), date seed flour (Ambigaipalan et al. 2015), royal jelly (Maqsoudlou et al. 2019), and mung bean protein (Xie et al. 2019).

Despite the multitude of mentioned advantages, challenges such as low bioavailability, instability, hydrophobicity, bitter taste, incompatibility, and reaction with other food compounds (e.g., metal ions) are considered among the factors limiting the use of bioactive peptides in the fortification and production of functional foods (McClements 2015). A method to reduce these disadvantages is the stabilization of peptides using different lipid-based nanodelivery systems include solid lipid nanoparticles (SLN), nanostructured lipid carriers (NLC), nanoemulsions, lipid-polymer hybrid nanoparticles and nanoliposomes (Safaeian Laein et al. 2023).

Nanoemulsions are promising nanocarriers with good stability, optical transparency, rheology and other functions correlated with innovative technologies to be applied in food and beverage products (McClements and Jafari 2018). SLNs are composed of solid lipids or in combination with surfactants that allow long-term release, less leakage and more stability of the system (Shtay et al. 2018). NLCs are composed of a liquid lipid mixed with solid lipids, furthermore, due to the disorganized structure of solid and liquid lipids this carrier can accommodate several compounds and enable a controlled release trend (Safaeian Laein et al. 2023).

Among lipid nanodelivery systems, nanoliposomes are food-grade carriers with the ability to simultaneously load several types of bioactives with different solubility (Tamjidi et al. 2013; Katouzian and Jafari 2016). These properties led to the use of nanoliposomes for encapsulation of various anionic and cationic peptides including whey (Mohan et al. 2018), sheep whey hydrolysates (Corrêa et al. 2019), rainbow trout (Ramezanzade et al. 2017), salmon protein (Li et al. 2015), white croaker (da Rosa Zavareze et al. 2014), flaxseed (Sarabandi and Jafari 2020a), collagen of bream scales (Mosquera et al. 2014), and fish gelatin peptide fractions (Hosseini et al. 2017); as well as to evaluate the structural properties, stability, and maintaining the biological activity of loaded peptides.

Legumes are known as a rich source of proteins, energy, carbohydrates, fibers, vitamins, phenolic compounds, and minerals in the human diet. So, legumes with a high protein content (about 21–29%) are of paramount importance (Medina-Torres et al. 2016). Among these sources, the white bean (*Phaseolus vulgaris*) is specifically important in the diet of people around the world. According to the statistics of the Food and Agriculture Organization (FAO) of the United Nations, the annual global production of this product reached > 26 million tons. Beans contain a multitude of bioactive compounds that play many physiological roles. There are also some inhibitor compounds of protein-digesting enzymes, some oligosaccharides, and some other anti-nutritional compounds (e.g., phytic acid, lectin, and tannins) in their composition (Wani et al. 2015).

Therefore, the abundance and economy of legumes can raise them as a natural source of protein for the production of bioactive peptides (especially for vegetarians). To the authors' knowledge, no comprehensive research has so far investigated the extraction and production of bioactive peptides and their loading into nanoliposomes from navy bean. The bitterness of these peptides is one of the most important challenges that make their use difficult. Therefore, identifying the amount of bitter AAs (BAA) can be useful in designing and selecting the optimal nano-system (with the highest encapsulation efficiency, physical stability and controlled release).

Therefore, the objectives of this study were:(i) enzymolysis of proteins extracted from white beans with alcalase and pepsin enzymes, (ii) evaluation of AA composition of protein and hydrolysates, (iii) effects of the enzyme type and hydrolysis time on the degree of hydrolysis (DH), AA composition (essential, hydrophobic, antioxidant),antioxidant indices (including scavenging of DPPH, ABTS, OH free radicals, reducing power, chelating Fe^2+^ and Cu^2+^ ions), (iv) evaluation of the structural characteristics (amide regions, secondary structures and changes in functional groups) of the primary proteins and the resulting hydrolysates by the Fourier-transform infrared (FTIR) spectrometry, finally(v) effects of the hydrolysate type and DH of peptides on physical properties, stability, encapsulation efficiency during storage, antioxidant activity, chemical structure, and morphology of nanoliposomes.

## Materials and methods

Navy-beans were purchased from a local market. The chemicals including alcalase 2.4 L. (serine-protease, *Bacillus licheniformis*) and pepsin (porcine gastric mucosa) enzymes, DPPH (1,1-Diphenyl-2-picrylhydrazyl), ABTS (2,2′-azino-bis (3-ethylbenzothiazoline-6-sulfonic acid) diammonium salt), TCA (trichloroacetic acid), comasi brilliant blue (G250), bovine serum albumin (BSA), thiobarbituric acid (TBA), and cholesterol were purchased from Sigma–Aldrich (St. Louis, MO, USA). Tween-80 and ferrous chloride were purchased from Merck (Darmstadt, Germany). Soybean phospholipids (PHOSPHOLIPON 85) and Alpha-deoxyribose were bought from Lipoid (Germany), and Fluka (Stockholm, Sweden), respectively. Other chemicals had an analytical grade.

### Production of protein concentrate

Proteins were extracted according to the method of Moghadam et al. (2020) with some modifications. Ground beans (Perten 3100, Germany) were first defatted by hexane (1:5w/v ratio) for 4 h. Then, the resulting flour was mixed with sodium chloride solution (0.33 M, pH = 9.5) at a ratio of 1:10 w/v for 2 h, centrifuged at 5000 × g for 20 min (Hanil, combi 514R, Korea). The pH of the supernatant was adjusted to 4 with 1 N hydrochloric acid, and then the precipitated proteins were freeze-dried (Christ, Germany) and stored at − 18 °C.

### Enzymatic hydrolysis

Navy-bean proteins were dissolved at a concentration (w/v) of 5% in 0.2 M phosphate buffer (pH = 8) for 30 min. Then alcalase and pepsin enzymes were added to the solution at an enzyme to substrate (w/w) ratio of 2%. Temperature and pH were adjusted for alcalase (50 °C, pH = 8.5) and pepsin (30 °C, pH = 2) in their optimal functional conditions. The reaction medium (in time intervals of 50 to 300 min) was placed in a water bath at 90 °C for 15 min after the completion of the hydrolysis process to deactivate the enzyme activity. The final solution was centrifuged at 5000 rpm for 10 min, then the separated supernatant was lyophilized, and stored at − 20 °C until use (Akbarbaglu et al. 2022).

### Composition of amino acids

The AA composition of hydrolysates was determined using the method of Alashi et al. (2014) by an HPLC device equipped with a reverse phase column. First, each of the hydrolysates was digested in 6N hydrochloric acid at 110 °C for 24 h. Free AAs were also determined after dissolving each of the hydrolysates in double-distilled water.

### Degree of hydrolysis and antioxidant properties

In this study, the effects of process time and enzyme type on DH, scavenging of DPPH, ABTS, OH free radicals, reducing power, and chelating Fe^2+^ and Cu^2+^ ions were determined according to the methods described by Akbarbaglu et al. (2023); briefly described in supplementary materials.

### Nanoencapsulation of peptides

Nanoliposomes were produced by the thin-layer hydration method according to Sarabandi and Jafari (2020a, b). An ethanol solution (10 mL) was prepared from lecithin (0.09 g) and cholesterol (0.01 g), and a thin film was produced with a rotary evaporator (Laborota 4002, Heidolph, Germany) in optimal conditions (60 rpm, 70 °C). Then, the resulting film was hydrated with a 10 mL of peptide solution (5 mg/mL), and particle size was reduced in 10 cycles (1 min on and 1 min off) using an ultrasound probe (UP200H, Hielsher, Germany).

### Determination of average particle size, PDI, and zeta potential

The values of these indicators were determined in nanoliposomes diluted with distilled water (100-fold) using a dynamic light scattering (DLS) system (NanoSizer 3000, Malvern Instruments, UK) at 25 °C and an angle of 90°.

### Evaluation of encapsulation efficiency (EE)

EE was calculated using the ultrafiltration method, with molecular weight (Mw) cut off = 30 kDa (Millipore, UK), and determining the amount of unloaded peptides compared to the total amount. Each sample of liposome (2.0 mL) was transferred to the filter and centrifuged at 2500 g for 10 min. The amount of free peptides was estimated by the method of Bradford (1976), and EE was reported as a percentage (Sarabandi and Jafari 2020a).

### Physical stability

The effects of peptide type, hydrolysis time, and temperature were evaluated on the physical stability and EE of liposomes during storage. To this end, 2 mL of each liposome sample was transferred to microtubes and incubated at ambient (25 °C) and refrigerated (4°C) temperatures for 28 days. Changes in average particle size and EE were measured according to described methods (Li et al. 2015).

### Antioxidant activity of nanoliposomes

In this study, the effects of peptide type and hydrolysis time on the antioxidant activity of nanoliposomes (DPPH free radical scavenging) were examined according to the method of Mosquera et al. (2014).

### FTIR spectroscopy

The effects of enzymolysis on the protein structure and nanoencapsulation of peptides were evaluated based on the chemical structure of nanoliposomes. Freeze-dried nanoliposomes were mixed with potassium bromide (KBr) at a ratio of 1:100 and made into discs by a pressing device. Finally, FTIR data of the original and hydrolyzed proteins as well as empty and loaded nanoliposomes were recorded at a frequency of 4000–400 cm^−1^ using an FTIR spectrophotometer (Shimadzu 8400, Japan) (Dutta et al. 2022).

### Morphological characteristics

First, 1–2 drops of diluted nanoliposome (50 times with distilled water) were poured on a lab slide and dried at room temperature. Then, the samples were coated with a thin layer of gold, and the morphological characteristics were evaluated under 25kV accelerating voltage with a scanning electron microscope (SEM) (Sarabandi and Jafari 2020a).

### Statistical analysis

Data were analyzed statistically with SPSS 19.0 software (SPSS Inc., Chicago, IL) using one-way analysis of variance (ANOVA). All tests were performed in triplicate. The means were compared using Duncan's test at a significance level of 5%.

## Results and discussion

The results of various researches have shown that the effects of the nature, Mw, electric charge, composition of AAs or DH of peptides on the nutritional value, biological activities and properties of nanoliposomes are not the same (Ramezanzade et al. 2017; Mohan et al. 2018; Corrêa et al. 2019). Therefore, it is necessary to investigate the characteristics of nanoliposomes under the influence of the type and DH of the produced peptides for a better understanding of their effects.

### Degree of hydrolysis and composition of amino acids

**Fig. 1:**
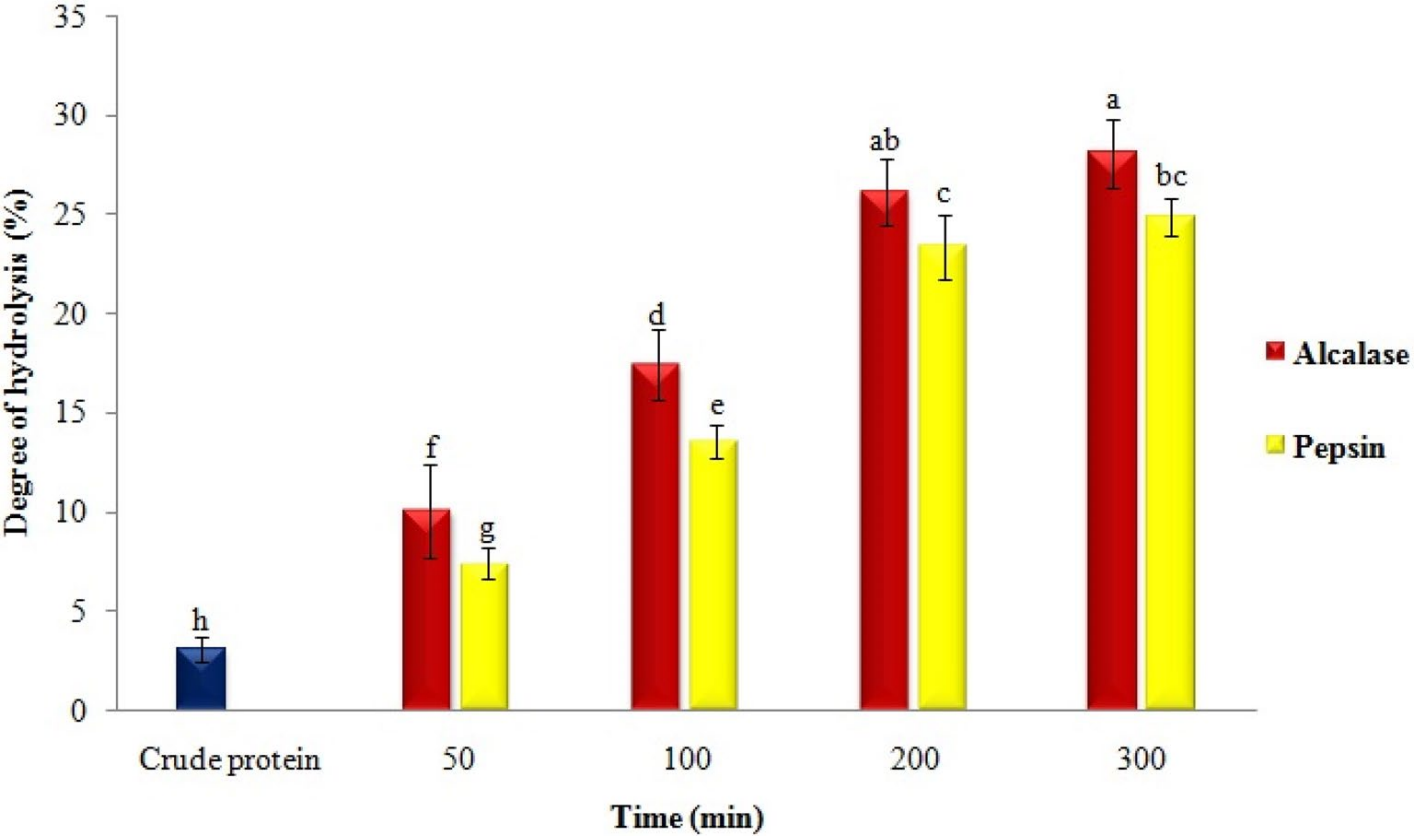
Effect of enzyme type and enzymolysis time on the degree of hydrolysis of navy-bean protein

In the first stage, the effects of enzyme type and hydrolysis time were investigated on DH and the composition of AAs (Fig. 1, Table 1). The DH and breakdown of peptide bonds in navy-bean proteins were affected by the study variables. The maximum value of DH was obtained for alcalase hydrolysates (~ 28%) and pepsin (~ 24%) after hydrolysis for 300 min. However, samples obtained after 200 min were not significantly different from those acquired after 300 min. Moreover, glutamic, aspartic, arginine, glycine, cystine, proline, alanine, valine, and leucine AAs comprised the most AA composition of protein or hydrolysates. The amounts of hydrophobic and antioxidant AAs for alcalase and pepsin hydrolysates were respectively about 38.5 and 16% of the total amount. The AA contents, along with Mw of peptides, were affected by DH and the type of enzyme.

**Table 1:** Effect of enzyme type and enzymolysis time on the amino acid (mg/g) composition of navy-bean proteins and its hydrolysates

Amino acid	Crude protein	Hydrolysate	
		Alcalase	Pepsin
Aspartic acid	85.3	86.9	89.1
Glutamic acid	104.5	106.3	108.4
Histidine	21.2	23.7	22.1
Serine	46.3	40.1	41.5
Arginine	40.7	38.6	37.2
Glycine	28.1	29.7	28.4
Threonine*	35.3	36.4	37.8
Alanine	30.2	32.1	29.9
Tyrosine	31.5	35.6	32.7
Methionine*	8.9	9.2	9.1
Valine*	40.6	39.1	40.2
Cysteine	80.2	77.9	79.6
Proline	35.3	34.3	35.7
Phenylalanine	44.8	47.3	46.2
Isoleucine*	36.4	37.9	37.4
Leucine*	64.9	65.2	67.2
Lysine*	47.3	50.2	47.9
Tryptophan*	6.3	8.9	6.8
HAA	298.2	308.7	305.2
AAA	115.2	127.6	118.6
BAA	240.3	248.6	245.7
TAA	787.8	798.5	799.2

^*^Essential amino acids; Hydrophobic amino acids (HAA) = Ala, Val, Ile, Leu, Tyr, Phe, Trp and Met; Antioxidant AAs (AAA) = Trp, Met, His, Tyr and Lys; Bitter AAs (BAA) = Trp, Ile, Leu, Val, Phe and Lys. Total AAs (TAA)

In a similar study, the DH of proteins from the grass turtle was affected by pH, temperature, enzyme concentrations, and reaction time. Furthermore, increasing the DH elevated the release of antioxidant and hydrophobic free AAs (Islam et al. 2021). In another study, the *Torreya grandis* meal protein was hydrolyzed with pepsin, trypsin, alcalase, and papain enzymes. The results showed that the highest (~ 10%) and the lowest (~ 4%) DH belonged to proteins hydrolyzed with alcalase and papain, respectively (Luo et al. 2021).

### Biological properties of peptides

#### Antioxidant activity

**Fig. 2:**
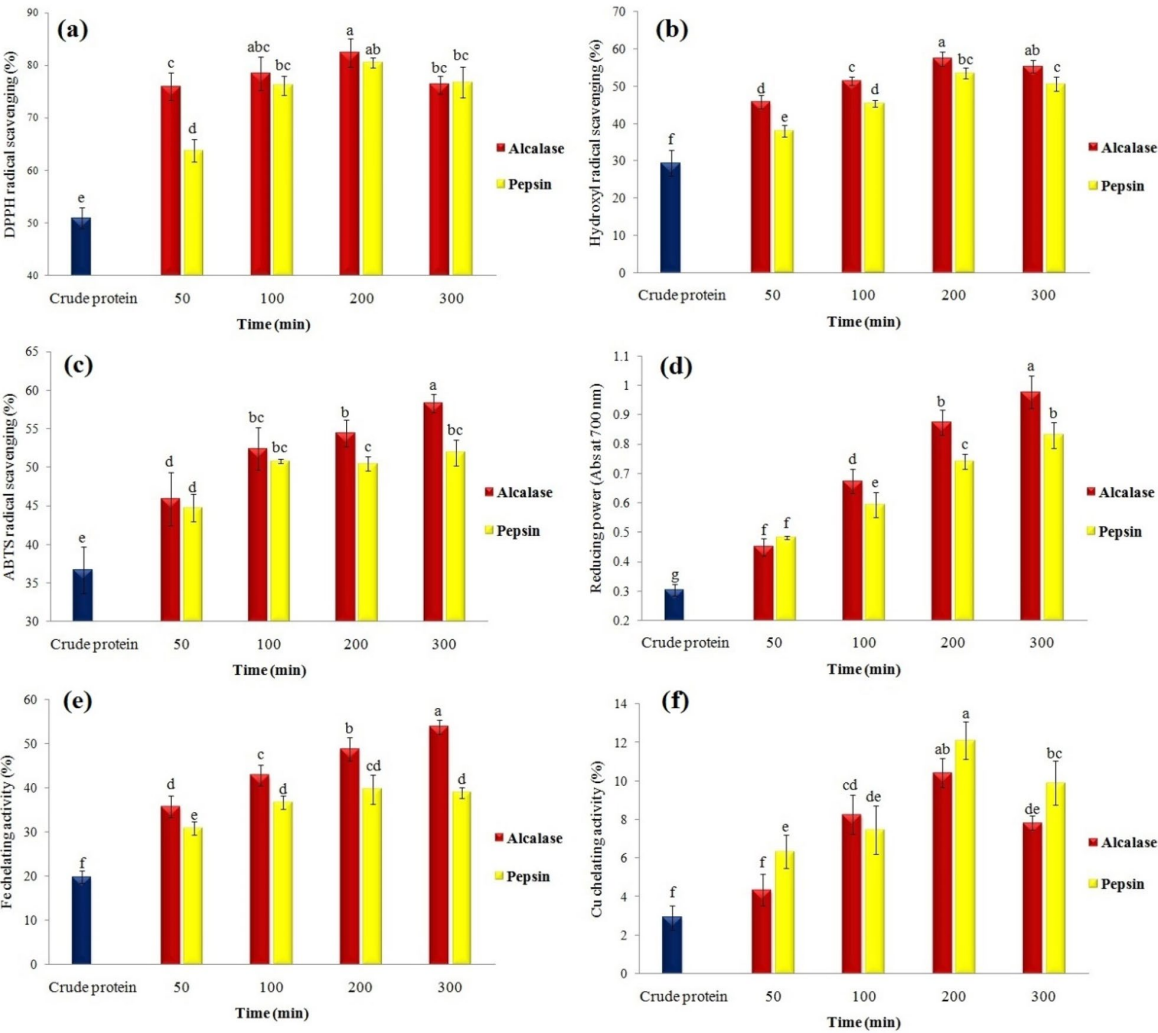
Effect of enzyme type and enzymolysis time on **a** DPPH, **b** Hydroxyl, **c** ABTS radical scavenging, **d** reducing power activity, **e** Fe chelating, and **f** Cu chelating activity of navy-bean proteins

Given the action mechanism of peptides in free radical scavenging, the effects of enzyme type and DH on various antioxidant indices and chelating properties of white bean hydrolysates were investigated in this stage. The results indicated that DPPH/OH free radicals scavenging were influenced by the study variables (Fig. 2a, b). For example, DPPH free radical inhibition increased from about 50% to 78% and 76% after the enzymolysis of white bean proteins with alcalase and pepsin, respectively, for 100 min; but the antioxidant activity of proteins was not affected by increasing the hydrolysis time. Additionally, similar results were observed regarding the OH radical scavenging test. The value of this index reached the maximum value for alcalase (57%) and pepsin (53%) hydrolysates after 200 min of hydrolysis (Fig. 2b).

Also, the antioxidant activity of white bean peptides was evaluated in inhibiting ABTS free radical and their reducing power (Fig. 2c, d). The maximum level of ABTS free radical inhibition (58%) was measured in the samples obtained from hydrolysis with alcalase (after 300 min). However, enzymolysis with pepsin for > 100 min did not affect the value of this index significantly. On the other hand, the reducing power of peptides was directly related to the hydrolysis time. After 300 min of enzymolysis, the value of this index increased from about 0.3 to 0.97 and 0.83% for the hydrolysates obtained from alcalase and pepsin, respectively (Fig. 2d). These findings suggest different functions of enzymes in the release of antioxidant and reactive AAs. These changes can be attributed to differences in DH, Mw, and, finally, the release of hydrophobic and antioxidant free AAs with proton-donating or electron-donating abilities (Shahidi and Zhong 2015).

Antioxidant properties are influenced by the type and composition of AAs. In this regard, some factors are effective: (i) the difference in the antioxidant activity of peptides can be attributed to the difference in the performance of enzymes in breaking peptide bonds, release, composition and sequence of AAs (hydrophobic, aromatic, sulfur-containing and nucleophilic types) of peptides (Lima et al. 2019); (ii) the increase of AAs with a positive charge increases the inhibition of free radicals. For example, lysine, arginine and histidine play a key role in the antioxidant activity of peptides (Song et al. 2020); (iii) the type of AAs released (e.g. electron/hydrogen donating activity, solubility, polarity and electrical charge) can be effective in their function to convert lipophilic (such as DPPH) and hydrophilic (such as ABTS) free radicals into stable forms (Bozkurt et al. 2021).

The results of another research in terms of the enzymolysis of black bean proteins using pepsin and alcalase on the antioxidant activity of hydrolysates indicated different effects of enzymes. Besides, the inhibition of ABTS free radical in alcalase-produced hydrolysates was > the samples resulting from pepsin (do Evangelho et al. 2017). An increase in the antioxidant activity of mung bean protein (Xie et al. 2019), walnut (Moghadam et al. 2020), canola pulp (Alashi et al. 2014), date seed (Ambigaipalan et al. 2015), *Spirulina* (Akbarbaglu et al. 2022), flaxseed (Sarabandi and Jafari 2020a), and walnut (Moghadam et al. 2020) after enzymolysis with different proteases was reported in similar studies.

#### Chelating activity of metal ions

Another challenge related to the storage of food products (especially with high fat) is the presence of metal ions and their role in the accelerated oxidation of fatty acids (Akbarbaglu et al. 2023). Therefore, production of natural chelating compounds and their use in food formulations is of paramount importance in the reduction of lipid oxidation risks. In this research, the effects of enzymolysis times of white bean proteins with different enzymes were investigated on the chelation of Fe^2+^ and Cu^2+^ ions (Fig. 2e, f). The results indicated significant effects of the above variables on the inhibition of metal ions. For example, the highest Fe^2+^ ion chelating activity (~ 54%) was obtained after 300 min of enzymolysis with alcalase while it was not affected by hydrolysis with pepsin for > 100 min. However, the peptides obtained from enzymolysis of white bean proteins reached the highest Cu^2+^ ion chelating activity (about 12% and 10% for alcalase and pepsin, respectively) after 200 min. The effect of enzymolysis on the chelation of metal ions can be attributed to the higher availability of carboxylic and amino groups resulting from the release of acidic (aspartic and glutamic) or basic (arginine and lysine) AAs, as well as the imidazole ring in histidine (Martínez-Palma et al. 2015). An increase in metal ion chelating activity after enzymolysis of grass turtle proteins (Islam et al. 2021), *Spirulina* (Akbarbaglu et al. 2022), flaxseed (Sarabandi and Jafari 2020a), phaseolin protein (Carrasco-Castilla et al. 2012), and mung bean (Liu et al. 2022) was reported in similar studies.

### Characterization of peptide-loaded nanoliposomes

#### Mean particle size, PDI, and zeta potential (ZP)

At this stage, the effects of peptide type and DH were investigated on average particle size, polydispersity index (PDI); factors affecting the stability, release, and EE (Hosseini et al. 2017). The average particle size and PDI of nanoliposomes were in the range of 82–116 nm and 0.23–0.35, respectively. The results showed that these indices increased after loading nanoliposomes with peptides (and also at higher DH) (Fig. 1). These findings might have resulted from the placement of peptides in the inner region and, especially, the monolayer structure of vesicles (Sarabandi and Jafari 2020a). Furthermore, the obtained PDI values indicated a narrow particle size distribution and a homogeneous system (Ramezanzade et al. 2017; Dutta et al. 2020). In a similar study, the effect of different concentrations (1–10 mg/mL) of fish gelatin hydrolysate was examined on the size of liposomes; particle size (from 134 to 621 nm) and PDI (from 0.27 to 0.49) increased at higher concentration of loaded peptides (Hosseini et al. 2017). Increases in particle size and PDI in nanoliposomes after loading with whey peptides (Mohan et al. 2016), orange core (Mazloomi et al. 2020), rainbow trout skin (Ramezanzade et al. 2017), and tilapia viscera (Sepúlveda et al. 2021) were also reported in other investigations.

As an index affecting the electrostatic stability of particles in the system, ZP examination revealed that it was influenced by the type of peptide and DH (Fig. 1, Table 1). Its value in the empty nanoliposome (− 20 mV) changed to − 15.9 mV and − 14.1 mV after loading the peptides obtained from enzymolysis with alcalase and pepsin, respectively, for 300 min. The reason can be the differences in Mw, type of peptide, and distribution of AAs (Ramezanzade et al. 2017). Also, placement of AAs and some peptides in the membrane and around liposomal particles can lead to changes in ZP (Corrêa et al. 2019). In different investigations, ZP of nanoliposomes loaded with white-mouth croaker peptides (da Rosa Zavareze et al. 2014), sheep whey (Corrêa et al. 2019), whey peptide fractions (Mohan et al. 2016) were reported to be − 5.5, − 16.7, and − 64–72 mV, respectively. The negative charges in peptides and empty nanoliposomes can be attributed to the presence of free AAs (e.g., glutamic and aspartic acids) and phosphate groups, respectively (Corrêa et al. 2019). Different indices, e.g. electric charge, the concentration of peptides, surface hydrophobicity, and the hydrolysate type, are factors that affect the physicochemical properties and stability of liposomal systems (Sarabandi and Jafari 2020a).

#### Encapsulation efficiency

EE was evaluated as an indicator of the carriers in loading bioactive peptides and thus their protection. The values of EE were 80–86% and 81–91%for peptides derived from alcalase and pepsin, respectively (Table 2). Among different peptides, the highest EE belonged to hydrolysates obtained from 200 min enzymolysis. Besides, a higher loading capacity was observed for the peptides obtained by hydrolysis with pepsin than the samples produced with alcalase. The reaction between peptides with monolayer membrane as well as phosphate groups can be effective factors on EE. Although other factors such as carrier particle size, concentration, molecular weight and electrical charge of peptides can also affect the encapsulation efficiency (Li et al. 2015; McClements 2015; Sarabandi et al. 2019). In this study, changes in ZP can be seen as a result of the presence of peptide factors with more positive charges in the samples produced with pepsin. Electrostatic interactions are expected to play a major role in the interplay between peptides and the liposomal structure. The difference in electricity charge enables these peptides to react with the negatively charged regions of the liposome. This is while the electrostatic repulsion between groups with the same charge leads to a decrease in EE (Mohan et al. 2018). Nanoliposomes loaded with Alc-Pep300 had the lowest EE, due to the faster release rate of peptides with less MW from the liposomal structure during preparation and storage. The differences in the size, Mw, and composition of AAs can be considered as the reasons affecting EE (Sarabandi and Jafari 2020a).

**Table 2:** Physicochemical properties and encapsulation efficiency (EE) of nanoliposomes loaded with navy bean protein hydrolysates

Treatments	Size (nm)	PDI	Zeta potential (mV)	EE (%)
Blank	82.9 ± 1.9^f^	0.23 ± 0.01^ g^	− 20.1 ± 1.1^ g^	–
Alc-50	96.3 ± 2.3^e^	0.38 ± 0.01^a^	− 18.5 ± 0.8^f^	79.8 ± 1.8^e^
Alc-100	103.1 ± 1.1^d^	0.29 ± 0.01^ef^	− 17.6 ± 0.6^ef^	83.4 ± 1.2^c^
Alc-200	107.8 ± 1.7^bc^	0.32 ± 0.01^c^	− 15.8 ± 0.6^ cd^	85.9 ± 1.5^b^
Alc-300	104.3 ± 1.3^ cd^	0.33 ± 0.01^c^	− 15.9 ± 0.5^ cd^	80.3 ± 1.3^d^
Pep-50	95.1 ± 1.2^e^	0.31 ± 0.01^de^	− 17.1 ± 0.8^de^	81.4 ± 1.4^ cd^
Pep-100	110.2 ± 4.3^abc^	0.28 ± 0.01^f^	− 15.2 ± 0.7^bc^	86.6 ± 1.8^b^
Pep-200	116.1 ± 7.2^a^	0.32 ± 0.01^ cd^	− 13.4 ± 0.5^a^	90.7 ± 1.5^a^
Pep-300	113.8 ± 5.9^ab^	0.35 ± 0.01^b^	− 14.1 ± 0.4^ab^	83.4 ± 0.9^c^

Data are presented as mean ± standard deviation (*n* = 3) and values denoted by different letters within each column are significantly different (P < 0.05)

In a similar investigation, antioxidant peptides from sheep whey were hydrolyzed and encapsulated inside nanoliposomes, with an EE of about 48%. The authors attributed the reduced value of EE to the repulsive forces between the anionic head of phospholipids and negatively charged peptides (Corrêa et al. 2019). In another study, however, the EE of fish gelatin hydrolysate decreased from 84% at a concentration of 1 mg/mL to 28% at a concentration of 10 mg/mL (Hosseini et al. 2017). In another report, encapsulation of cationic and anionic peptide fractions from whey hydrolysates yielded EE = 92 to 88% for these fractions, respectively (Mohan et al. 2018).

#### Physical stability and EE retention

**Fig. 3:**
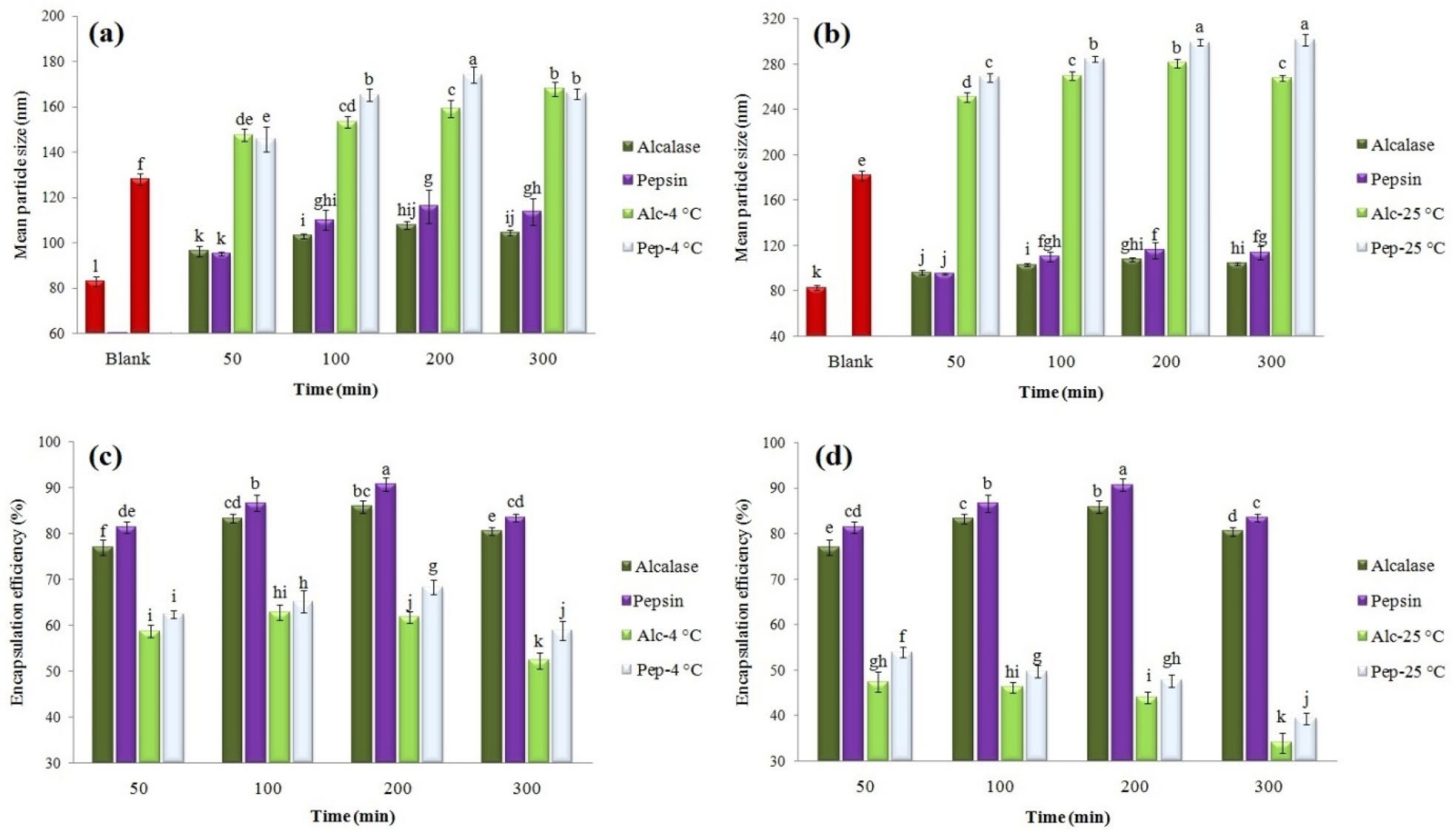
Effect of hydrolysate type and storage temperature on the **a**, **b** mean particle size and **c**, **d** encapsulation efficiency of loaded nanoliposomes

Here, the effect of loaded peptide type was investigated on the physical stability (changes in particle size) of nanoliposomes stored at 4 °C (Fig. 3a) and 25 °C (Fig. 3b). The size of control nanoparticles increased from 83 to 128 nm (at refrigerated) and 182 nm (at ambient temperatures) after 4 weeks storage. A similar trend was observed for peptide-loaded nanoliposomes. For example, the size of H-Alc200-containing nanoliposomes increased from about 107 nm to 159 and 280 nm under the same conditions. Overall, no significant differences were observed among the liposomes loaded with different peptides, but ambient temperature significantly influenced the physical stability of the system. Maintaining membrane strength, lower molecular mobility, and the reduced accumulation of nanoparticles at refrigerated temperature can be considered the reasons for these findings (Tan et al. 2016; Sarabandi and Jafari 2020a).

One of the determining indices in the stability of loaded bioactives is the retention of EE, which was influenced by the type of peptide (DH) and the storage temperature of nanoliposomes. As such, an increase in DH in the ambient temperature led to a higher release rate and, consequently, a decrease in EE. These changes were specifically higher in nanoliposomes kept at ambient temperature (Fig. 3c, d). For instance, EE of H-Alc200-containing nanoliposomes decreased from about 85% to 61% and 44% after 28 days storage in refrigerated and ambient temperatures, respectively. The continuous release of loaded peptides inside nanoliposomes can be ascribed to the controlled release mechanism (Hosseini et al. 2017). However, these findings indicated the effect of peptide type (in terms of DH and the type of enzyme) and, especially, the storage temperature on the stability of the membrane and the release rate of peptides from nanoliposomes.

Concerning the effect of peptide type, the electrostatic repulsion between anionic peptides and the phospholipid head of the carrier can be possible factors affecting the reduction of EE (Mohan et al. 2018). The effect of refrigerated temperature on the retention of EE can also be attributed to the preservation of membrane hardness, reduced permeability of the structure, and the physicochemical stability of nanoliposomes (da Rosa Zavareze et al. 2014). Similar results were reported in nanoliposomes loaded with salmon (Li et al. 2015) and flaxseed (Sarabandi and Jafari 2020b) peptides, in which the reduction of particle accumulation or formation of flocculates were observed in refrigerated storage conditions. In another study, an increase in the size of nanoliposomes loaded with sheep whey peptides was observed from 167 to 251 nm after 30 days of storage (Corrêa et al. 2019).

#### Effect of peptide type on the antioxidant activity of nanoliposomes

**Fig. 4:**
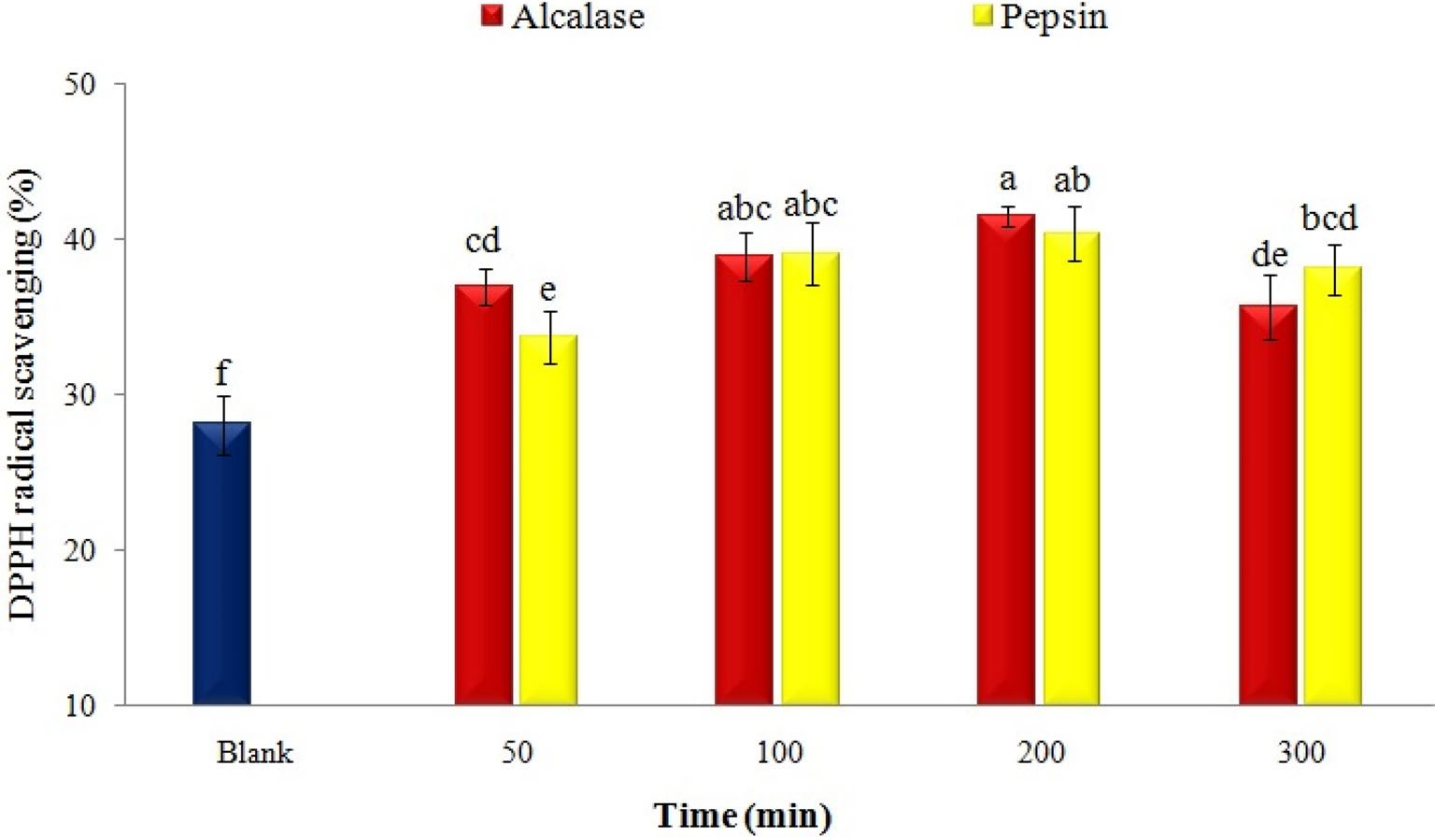
Effect of hydrolysate type on the antioxidant activity of loaded nanoliposomes

At this phase, the effect of peptide type and encapsulation process on the antioxidant activity and DPPH free radical inhibition of nanoliposomes was inspected (Fig. 4). The antioxidant activity of empty nanoliposome was about 28%. This finding suggests the hydrogen-donation ability of soy lecithin to DPPH free radicals and the antioxidant activity of the carrier (Li et al. 2022). DPPH free radical scavenging activity was about 65, 58, and 40% by soy lecithin with purities of 50, 70, and 98%, respectively (Li et al. 2022). The antioxidant activity of nanoliposomes increased after loading with bean peptides, and it was also affected by the type of peptides. For example, about 40% antioxidant activity was recorded for nanoliposomes loaded with H-Alc200 and H-Pep200. In other studies, the antioxidant activity of nanoliposomes loaded with algal extract (Savaghebi et al. 2020), marine fish peptides (Mosquera et al. 2014), flaxseed (Sarabandi and Jafari 2020a), and fish skin gelatin (Hosseini et al. 2017) were affected by the type, core concentration, and storage conditions.

#### Chemical structure of proteins, peptides and nanoliposomes

**Fig. 5:**
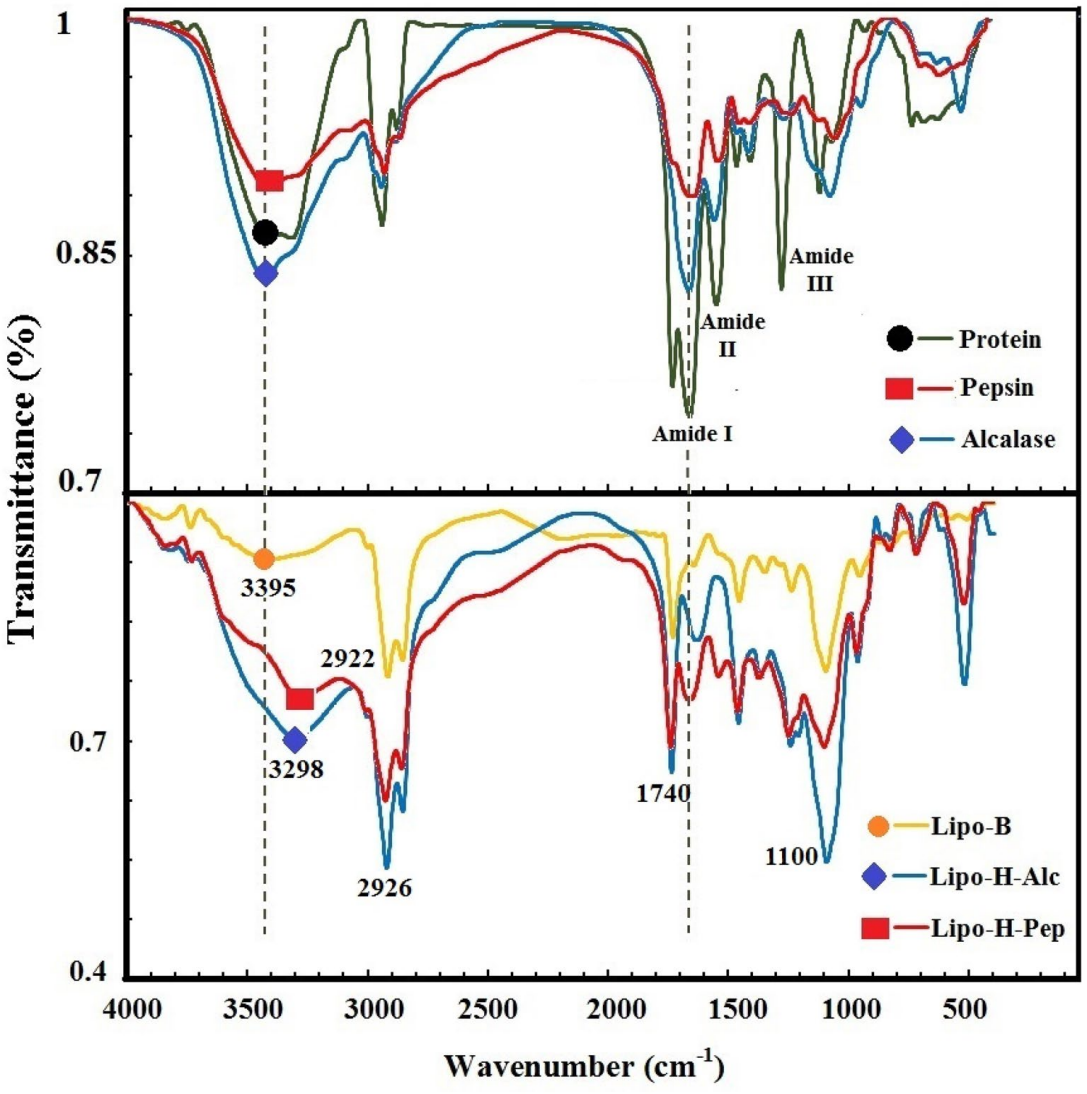
Effect of enzymolysis and loading peptides on chemical structures (FTIR) of protein and nanoliposomes

In this study, the effects of enzymolysis of white bean proteins and encapsulation of peptides were studied on the chemical structure of proteins and nanoliposomes (Fig. 5). FTIR spectroscopy is a non-destructive and precise technique to evaluate the chemical composition, structural arrangement, and also functional groups of various compounds. The main spectra for the primary proteins and peptides produced with alcalase and pepsin were 3419–3415 cm^−1^ (N–H stretching), 2928–2926 cm^−1^ (O–H stretching), 1647–1639 cm^−1^ (C = O stretching) related to amide I region, 1543–1539 cm^−1^belonging to N–H stretching, C-N deformation, and amide II vibrations, 1063–1052 cm^−1^ (C = O stretching), and 1617–621 cm^−1^ (N–H bending). Similar frequencies were observed in peptides obtained from enzymolysis of *Spirulina* algae (Akbarbaglu et al. 2022) and protein/collagen hydrolysates of a fish species (da Rosa Zavareze et al. 2014). Examination of the amide regions I and II indicates changes in the protein secondary structure after enzymolysis. Most of these changes result from alpha helix structures (1650–1658 cm^−1^) and random coils (1650–1640 cm^−1^). Changes and shifts in the peaks intensity can also be detected after hydrolysis. The lower intensity of the peak in the 1539–1543 cm^−1^ region (belonging to N–H bending and C-N stretching in the amide II region) in the hydrolyzed products is due to changes in the NH groups buried in the hydrophobic regions of the proteins (Poulsen et al. 2016).

Loading of peptides into nanoliposomes resulted in changes in the FTIR-spectra of the carrier, the most important one was the shift of 3395 cm^−1^ peak to 3298 and 3279 cm^−1^, respectively, after loading H-Alc200 and H-Pep200. These changes can be attributed to the formation of hydrogen bonds between the N–H and O–H groups in lecithin and peptides (da Rosa Zavareze et al. 2014). A shift and increase in the intensity of 2922 cm^−1^peak to 2926 cm^−1^ was also observed after loading peptides. These changes might be caused by the placement of peptides in the monolayer membrane or the formation of an ionic complex between them and phosphatidylcholine (Sarabandi and Jafari 2020a).

Another observed event was an increase in the peak intensity and its shift from 1795 to 1740 cm^−1^ after encapsulation, consequently, the formation of hydrogen bonds between the peptides and the carbonyl group of phosphatidylcholine. On the other hand, loading of peptides increased the peak intensity and shifted it from 1099 to 1100 cm^−1^. It shows a frequency of 1099 cm^−1^ (symmetric stretching vibrations of PO^2−^ in phospholipids). Also, the frequency of 959 cm^−1^ indicates the asymmetric stretching vibrations of the choline group (N^+^CH_3_) in the polar part of phosphatidylcholine (da Rosa Zavareze et al. 2014). The changes can be attributed to the formation of hydrogen bonds between the hydrogen atoms of peptides with phosphate groups, as well as the placement of peptides in the polar regions of nanovesicles (Ramezanzade et al. 2017).

#### Morphological characteristics of nanoliposomes

**Fig. 6:**
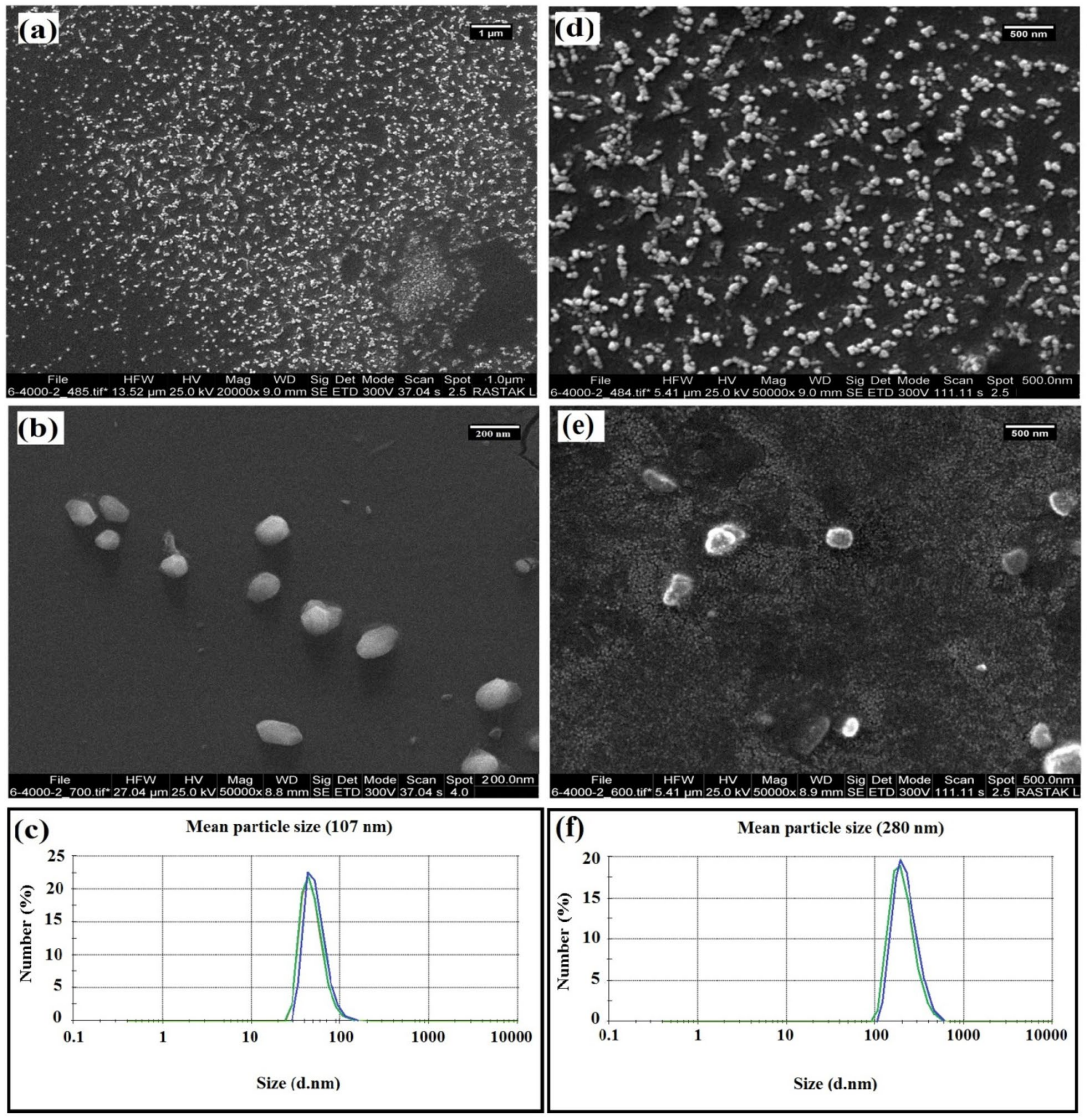
Morphological characteristics (SEM-images) and DLS-images of nanoliposomes loaded with Alc-200 after production **a**–**c** and storage for 28 days (d-f)

The morphology of liposomal H-Alc200-containing nanocarriers (as a sample) were evaluated after production (Fig. 6a, b) and storage (at ambient temperature)(Fig. 6c, d). SEM images depicted discrete particles with irregular structures and smooth surfaces. The dimensions of initial particles and a change in the particle size after storage were also visible (confirming the results of DLS).

Other investigations reported the production of vesicles with a spherical structure (Li et al. 2015), or particles with smooth surfaces and attached together (Sarabandi and Jafari 2020a), and integrated structures of spherical nanovesicles with a narrow size distribution (Nahr et al. 2019) after loading nanoliposomes with bioactive compounds.

## Conclusion

In this research, white bean protein was selected as a plant source for the extraction and production of bioactive peptides. Enzymolysis was performed with different proteases at a range of times. The evaluation of AA composition indicated that the peptides were rich in hydrophobic and antioxidant AAs. The enzyme type and hydrolysis time were factors affecting such indices as DH and antioxidant activities of peptides (free radical scavenging and chelating metal ions).Furthermore, the surface charge and DH of peptides influenced the physicochemical properties, EE, release rate, and stability of nanocarriers during storage. The evaluation of chemical structure (FTIR) suggested the successful loading of peptides into nanoliposomes (inner hydrophilic structure and bilayer membrane) and maintaining their antioxidant activity. Morphological features and changes in the particle size of nanocarriers were assessed with SEM images. Finally, white bean protein is a natural and rich source of bioactive peptides that can be used in food formulations after appropriate enzymolysis and nanoencapsulation.

### Supplementary Information

**Additional file 1.** Additional information.

## Data Availability

All data generated or analyzed during this study are included in this article.
