# Correction: Protection of navy-bean bioactive peptides within nanoliposomes: morphological, structural and biological changes

**DOI:** 10.1186/s40643-024-00733-z

**Published:** 2024-01-24

**Authors:** Nazila Zeynali Namdar, Leila Roufegarinejad, Ainaz Alizadeh, Narmela Asefi, Seid Mahdi Jafari, Khashayar Sarabandi

**Affiliations:** 1 Department of Food Science and Technology, Islamic Azad University, Tabriz Branch, Tabriz, Iran; 2 Department of Food Materials & Process Design Engineering, Gorgan University of Agricultural Sciences and Natural Resources, Gorgan, Iran; 3 Research Institute of Food Science and Technology (RIFST), Km 12 Mashhad‑Quchan Highway, 91895‑157‑356, Mashhad, Iran; 4 Halal Research Center of IRI, Iran Food and Drug Administration, Ministry of Health and Medical Education, Tehran, Iran

**Correction: Bioresources and Bioprocessing (2023) 10:87** 10.1186/s40643-023-00709-5

In this article (Namdar et al. 2023), the affiliation ‘Halal Research Center of IRI, Iran Food and Drug Administration, Ministry of Health and Medical Education, Tehran, Iran’ for the fifth author, Seid Mahdi Jafari was missing.
