# Supplementary material for: Protection of navy-bean bioactive peptides within nanoliposomes: morphological, structural and biological changes
Source: Bioresour Bioprocess. 2023 Dec 1;10(1):87. doi: 10.1186/s40643-023-00709-5 (PMC10992752; doi:10.1186/s40643-023-00709-5)
Supplement: Supplementary file 1 — Additional file 1. Additional information. [file 40643_2023_709_MOESM1_ESM.docx]

**DPPH radical scavenging**

An aliquot of 2 mL of peptide solution (40 mg/mL) was mixed with 2 mL of 0.2 mM DPPH ethanolic solution. Then, the obtained mixture was kept at dark for 30 min followed by centrifugation at 5,000 × g for 10 min and the absorption of supernatant was measured at 517 nm. The DPPH radical scavenging percentage was calculated by the following equation:

DPPH Inhibition (%) = [1- (Abs_sample_ /Abs _blank_)] × 100 (1)

**ABTS radical scavenging**

A solution containing both of ABTS (7 mM) and potassium persulfate (2.45 mM) was prepared, and after 12 h keeping in darkness, diluted with 0.2 M PBS (pH= 7.4) until its absorbance (at 734 nm) reduced to 0.70. Then, 20 µL of peptide solution (10 mg/mL) was added to 2 mL of this ABTS solution, vortexed for 10 s, and the absorbance of mixture recorded at 734 nm after reacting in darkness for 6 min.

**Hydroxyl radical scavenging**

A reaction mixture consisting of 0.2 mL of peptide solution (40 mg/mL), 0.5 mL of α-deoxyribose solution (10 mM), 0.2 mL of FeSO4-EDTA solution (10 mM), 0.9 mL of PBS (0.2 M, pH= 7.4) and 0.2 mL of hydrogen peroxide solution (10 mM) was incubated at 37°C for 1 h. Then, 1.0 mL of each of 1.0% TBA and 3% TCA solutions was added. The absorbance of final mixture, after placing in boiling water for 15 min and cooling in ice-bath, was recorded at 532 nm.

**Reducing power**

A mixture consisting of 0.5 mL of peptide (40 mg/mL) solution, 0.5 mL potassium ferricyanide (1%) and 0.5 mL of 0.2 M PBS (pH 6.6) was stored at 50°C for 20 min. After that, 0.5 mL of TCA solution (10%) was incorporated to the mixture and centrifuged (3,000 × g, 15 min). Subsequently, absorbance of a mixture of supernatant (1.0 mL), distilled water (1.0 mL) and 0.1% FeCl_3_ solution (0.2 mL) was read at 700 nm.

**Fe^2+^ chelating activity**

A reaction mixture consisting of 1 mL of peptide solution (40 mg/mL), 50 μL of iron (II) chloride solution (2 mM), 1.8mL of double distilled water and 0.1 mL of Ferrozine (5 mM) solution was vortex-mixed and after 10 min keeping at room temperature, its absorbance monitored at 562 nm.

**Cu^2+^ chelating activity**

At first, 1 mL of peptide solution (40 mg/mL) was mixed with 1 mL of CuSO_4_ solution (0.2 mM), and incubated at 25°C for 5 min. Then, 1 mL of TCA solution (10%) was added and centrifuged (2,000 × g, 10 min). Finally, a mixture of 2 mL of the supernatant, 20 μL of pyrocatechol-violet solution (0.1%) and 1 mL of pyridine solution (10%) was vortex-mixed and its absorbance monitored at 632 nm after 5 min incubation at room temperature.
